# The prevalence of short sleep duration and sleep disturbances among working-age adults in Poland – a secondary analysis

**DOI:** 10.3389/fpubh.2026.1812130

**Published:** 2026-05-15

**Authors:** Radosław Sierpiński, Mateusz Jankowski, Agnieszka Kamińska, Filip Raciborski

**Affiliations:** 1 Faculty of Medicine, Collegium Medicum, Cardinal Stefan Wyszynski University, Warsaw, Poland; 2 School of Public Health, Centre of Postgraduate Medical Education, Warsaw, Poland; 3 Department of Prevention of Environmental Hazards, Allergology and Immunology, Faculty of Health Sciences, Medical University of Warsaw, Warsaw, Poland

**Keywords:** employees' health, mental health, Poland, public health, workforce health, workplace health promotion

## Abstract

**Introduction:**

Sleep is essential for physical, mental and occupational functioning. This study aimed to assess the prevalence of short sleep duration and sleep disturbances among working-age adults in Poland as well as to identify factors associated with sleep disturbances.

**Methods:**

This study is a secondary analysis of data derived from a nationwide cross-sectional survey (December, 2024) on health-related behaviors among 5,006 working-age adults (18–64 years) in Poland. Questions on sleep health were analyzed. Short sleep duration was defined as less than 7 h per day.

**Results:**

In the study population (*n* = 5,006), 17.9% of respondents reported sleeping less than 7 h on weekdays: 21.7% for men and 14.1% for women (*p* < 0.001). Among the respondents, 6.5% reported sleeping less than 7 h on weekends: 8.0% among men and 4.9% among women (*p* < 0.001). Sleep disturbances were reported daily or almost daily during the past three months by 12.0% of respondents, a further 17.5% reported experiencing them a few times a week, and 22.5% a few times a month. Additionally, 15.8% reported sleep disturbances less than once a month, while 32.5% reported never experiencing them. There were significant differences (*p* < 0.05) by gender, age, educational level, place of residence, employment status, self-reported financial situation, and self-reported health status. In multivariable logistic regression model, female gender (*p* < 0.001), having primary (*p* < 0.001) or secondary education (*p* < 0.05), living in cities over 500,000 inhabitants (*p* < 0.01), poor financial situation (*p* < 0.001) and bad health status (*p* < 0.001) were significantly associated with higher odds of reporting sleep disturbances.

**Conclusions:**

This study revealed a relatively high prevalence of short sleep duration on weekdays among working-age adults. Findings from this study underline the need to strengthen public health interventions focused on sleep health. Interventions should be particularly targeted to males, older adults, those with primary or vocational education, and those of poor economic status.

## 1 Introduction

Sleep is essential for physical, mental and occupational functioning (1–3). Moreover, sleep also affects wellbeing, cognitive function and emotional regulation (2, 3). Insufficient or disturbed sleep is associated with increased risk of chronic diseases, including cardiovascular diseases, diabetes, obesity, mental health problems and reduced immune functioning (4, 5). In working-age adults, sleep disturbances lead to increased risk of workplace accidents, error rates, sickness absence and reduced productivity (4–6).

Research on sleep health are commonly focused on two indicators: sleep duration and sleep disturbances (7–10). In working-age adults, short sleep duration is commonly defined as sleeping less than 7 h per night, following the National Sleep Foundation's sleep time duration recommendations and other international health organizations (8). Numerous studies showed that sleeping less than 7 h per night is often associated with increased health risk due to insufficient time for rest and regeneration of physical and mental health during sleep (7–9). Sleep disorders are group of conditions that disrupts the quality, duration, patterns and timing of sleep (10). There are multiple medical conditions that may lead to sleep disturbances, including insomnia, obstructive sleep apnea, narcolepsy, brain and nerve conditions, obesity, heart diseases, cancers, diabetes, chronic pain (1–5, 8, 10).

Sleep health is a growing public health issue. The prevalence of sleep disorders is growing worldwide (11, 12). Moreover, sleep disorders are often underdiagnosed (12, 13). Working-age adults are of particular attention, as job organization, shift work, screen exposure, psychological and social stress may contribute to higher risk of sleeping disorders (10, 14). Moreover, insufficient duration and quality of sleep generates significant social and economic costs (15, 16). Within the lifestyle medicine framework, sleep is recognized as a fundamental pillar alongside physical activity, nutrition, and stress management, exerting a substantial influence on both health outcomes and overall wellbeing (3, 5, 8, 9). Moreover, sleep health is increasingly recognized in global public health initiatives, as modifiable determinant of health (17).

In Poland, there is a limited number of studies on sleep health (18–20). Most of the studies are focused on sleeping problems in selected populations of patients with chronic diseases (18, 19). Additional studies have examined sleep health in selected populations in Poland, including cohort studies and clinical samples. However, nationally representative data remain scarce. Moreover, sleep health is not implemented in major public health strategies in Poland. Understanding the prevalence and sociodemographic differences of short sleep duration and sleep disturbances may provide scientific evidence for public health interventions, workplace health promotion initiatives and occupational risk assessments (17, 21).

This study aimed to assess the prevalence of short sleep duration and sleep disturbances among working-age adults in Poland as well as to identify factors associated with sleep disturbances.

## 2 Material and methods

### 2.1 Study design and population

This study is a secondary analysis of data derived from a nationwide cross-sectional survey on health-related behaviors among working-age adults in Poland. The dataset was obtained from the National Center for Health Policy and Health Inequalities at Cardinal Stefan Wyszynski University, which conducted the survey titled “Health prevention and health inequalities” and made the anonymized data available for scientific use (22). The original survey was implemented under a contract with the Polish Ministry of Education and Science (Agreement No. MEiN/2023/DPI/2717 of 13/10/2023); consequently, the Center enables non-commercial, free-of-charge data sharing.

The study protocol was reviewed and approved by the Ethics Committee at the Medical University of Warsaw (decision No. AKBE/56/2025).

The original survey was carried out between 4 and 16 December 2024 on a nationally representative sample of 5,006 adults aged 18–64 years (working-age population), including both employed and non-employed individuals (22, 23). Therefore, the study population reflects the general working-age population rather than exclusively the employed workforce. A quota-based sampling strategy was applied, with sex, age, educational level, and size of place of residence as variables included in the sampling model. Demographic data published by the Statistic Poland were used to calculate the demographic structure of the study population (24). The final dataset was weighted to reflect the national distribution of Polish adults aged 18–64 years.

Data were collected by an external public opinion research company (ARC Rynek i Opinia) (25). Participants were recruited from the company's verified respondent pool and completed the questionnaire using the computer-assisted web interview (CAWI) method. The questionnaire was available online, via a secure dedicated system. All questions were mandatory in the survey system; therefore, no missing data were recorded. If someone refused to participate, another respondent meeting the same quota criteria was invited (replacement procedure). Participation was voluntary, and the informed consent was collected during the data collection process.

### 2.2 Measures

Three questions on sleep quality and duration were obtained from the original dataset for this study.

Sleep duration was assessed using the following questions: (1) During your typical workweek, at what time do you usually: Fall asleep (HH:MM) (hour-minute); Wake up (HH:MM) (hour-minute); (2) During a typical weekend (Saturday and Sunday), at what time do you usually: Fall asleep (HH:MM) (hour-minute); Wake up (HH:MM) (hour-minute). Following the National Sleep Foundation's sleep time duration recommendations, short sleep duration was defined as sleeping less than 7 h (8).

Sleep disturbances were assessed using the following question “In the last 3 months, have you experienced insomnia, trouble falling asleep, or waking up in the middle of the night and having trouble falling back asleep?,” with the following answers: yes, every day or almost every day; yes, a few times a week; yes, a few times a month; less than once a month; no.

This composite question was intended to capture common symptoms of sleep disturbances in population-based research. However, it does not allow differentiation between specific sleep disorders and may lead to heterogeneity in interpretation across respondents.

### 2.3 Data analysis

Data were analyzed with IBM SPSS Statistics v29 (IBM, Armonk, NY, USA). Categorical variables are presented as frequencies and proportions. Cross-tabulation with chi-squared tests were used to compare categorical variables.

Factors associated with sleep disorders - every day or almost every day were identified using multivariable logistic regression model. Predictors were entered as sets of dummy variables (0/1). Model performance was summarized using Cox–Snell R^2^ and Nagelkerke R^2^. All analyses, including descriptive statistics and multivariable regression models, were conducted using sampling weights to ensure national representativeness of the estimates. The strength of associations was presented with odds ratios (ORs) and 95% confidence intervals (95% CI). Statistical significance was set at *p* < 0.05.

## 3 Results

A total of 5,006 adults aged 18–64 years participated in the study (Table 1). The mean age was 41.8 years (SD = 12.6), and 50.0% of respondents were women. Detailed characteristics of the study population are presented in Table 1.

**Table 1:** Characteristics of the study population (*n* = 5006).

	*n* (%)
Gender	
male	2,506 (50.1)
female	2,500 (49.9)
Age group [years]	
18–24	541 (10.8)
25–34	989 (19.8)
35–44	1,326 (26.5)
45–64	2,150 (42.9)
Educational level	
primary or vocational	1,593 (31.8)
secondary	1,895 (37.9)
higher	1,518 (30.3)
Place of residence	
rural area	2,035 (40.6)
city < 100,000	1,582 (31.6)
city 100,000–499,000	789 (15.8)
city >= 500,000	602 (12)
Employment	
yes, full-time	2,863 (57.2)
yes, part-time	902 (18.0)
no	1,242 (24.8)
Self-assessment of financial situation	
we have enough for everything and we're saving for the future	999 (20.0)
we have enough for everything without any special sacrifices, but we're not saving for the future	977 (19.5)
we live frugally and therefore have enough for everything	1,804 (36.0)
we live very frugally to save for major purchases	678 (13.6)
we only have enough money for basic needs or we don't have enough money for even the cheapest food	547 (10.9)
Respondent's health compared to peers	
definitely better or slightly better	1,342 (26.8)
the same or hard to say	2,323 (46.4)
slightly worse	1,003 (20)
definitely worse	338 (6.7)
Sleep less than 7 h on weekdays	
yes	896 (17.9)
no	4,110 (82.1)
Sleep less than 7 h on weekends	
yes	324 (6.5)
no	4,681 (93.5)
Sleep disturbances in the last 3 months	
yes, every day or almost every day	603 (12.0)
yes, a few times a week	875 (17.5)
yes, a few times a month	1,109 (22.2)
less than once a month	789 (15.8)
no	1,629 (32.5)

**Figure 1:**
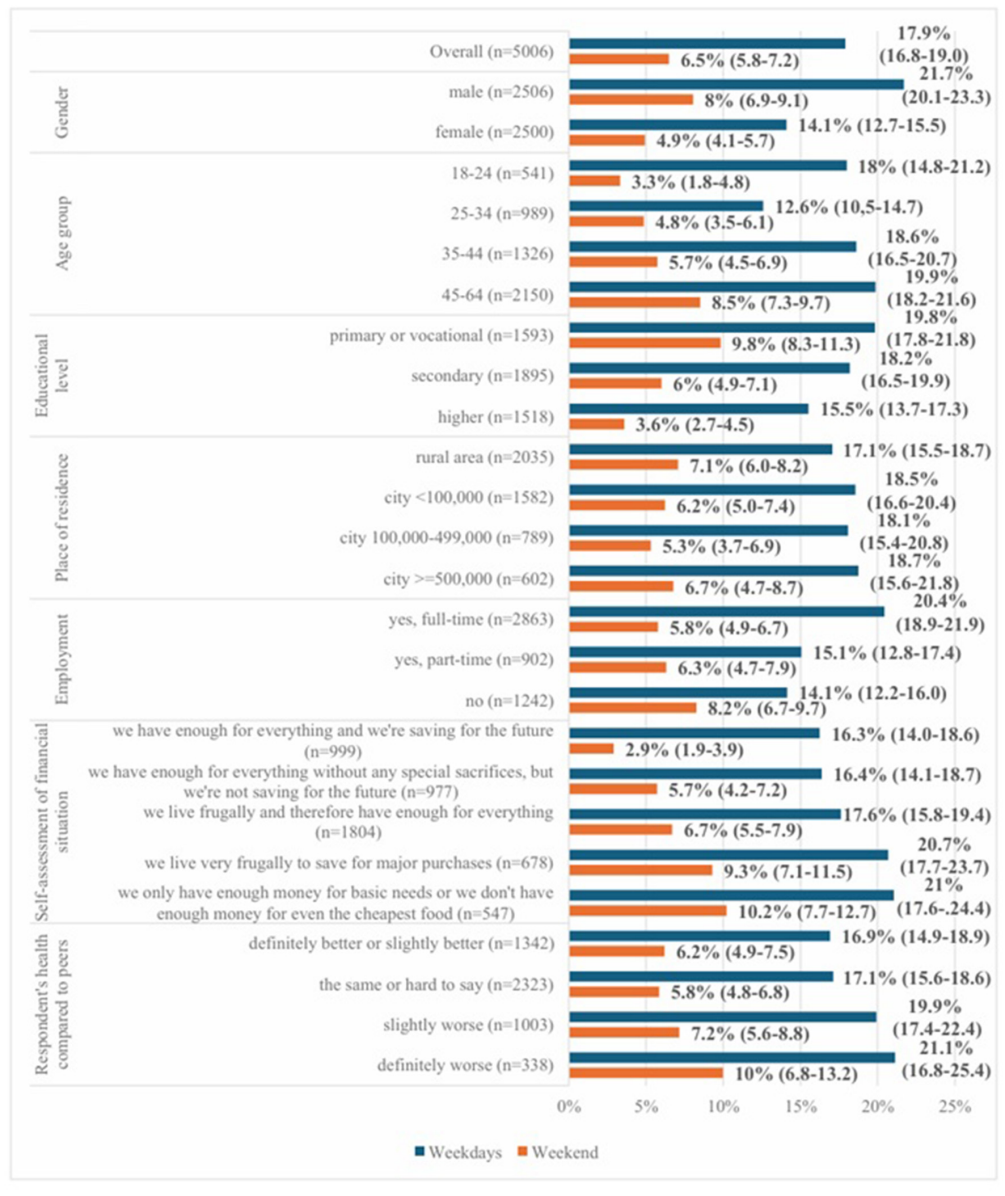
Percentage (along with 95% confidence intervals) of respondents reporting sleeping less than 7 h a night during the weekdays and on weekends (*n* = 5006).

The prevalence of short sleep duration (< 7 h) was 17.9% on weekdays and 6.5% on weekends. The median sleep duration was 8 hours on weekdays and 9 h on weekends. Sleep disturbances occurring daily or almost daily were reported by 12.0% of respondents. Short sleep duration on weekdays was more common among men, older adults, individuals with lower educational attainment, those employed full-time, and respondents reporting a poorer financial situation. In contrast, short sleep duration on weekends was less frequent and showed similar sociodemographic patterns (Figure 1).

Sleep disturbances were reported daily or almost daily during the past three months by 12.0% of respondents, a further 17.5% reported experiencing them a few times a week, and 22.5% a few times a month (Table 2). Additionally, 15.8% reported sleep disturbances less than once a month, while 32.5% reported never experiencing them. There were significant differences (*p* < 0.05) by gender, age, educational level, place of residence, employment status, self-reported financial situation, and self-reported health status (Table 2).

**Table 2:** Percentage of respondents reporting sleep disturbances in the last 3 months (*n* = 5,006).

In the last 3 months, have you experienced insomnia, trouble falling asleep, or waking up in the middle of the night and having trouble falling back asleep?						
	yes, every day or almost every day	yes, a few times a week	yes, a few times a month	less than once a month	no	*p*
Overall (*n* = 5,006)	12.0%	17.5%	22.2%	15.8%	32.5%	
Gender						
male (*n* = 2,506)	9.9%	15.4%	21.8%	16.0%	36.9%	< 0.001
female (*n* = 2,500)	14.2%	19.5%	22.6%	15.6%	28.2%	
Age group [years]						
18–24 (*n* = 541)	13.3%	16.6%	21.6%	18.3%	30.1%	< 0.01
25–34 (*n* = 989)	11.2%	21.0%	20.8%	15.4%	31.6%	
35–44 (*n* = 1,326)	9.6%	18.3%	23.9%	15.1%	33.1%	
45–64 (*n* = 2,150)	13.7%	15.5%	21.9%	15.7%	33.2%	
Educational level						
primary or vocational (*n* = 1,593)	15.7%	16.3%	20.0%	13.4%	34.7%	< 0.001
secondary (*n* = 1,895)	11.6%	18.7%	23.4%	15.0%	31.2%	
higher (*n* = 1,518)	8.8%	17.1%	22.9%	19.2%	31.9%	
Place of residence						
rural area (*n* = 2,035)	11.5%	16.4%	23.0%	15.0%	34.1%	< 0.05
city < 100,000 (*n* = 1,582)	12.4%	18.8%	20.8%	15.0%	33.0%	
city 100,000–499,000 (*n* = 789)	10.7%	18.5%	22.7%	18.3%	29.8%	
city >= 500,000 (*n* = 602)	14.8%	16.3%	22.1%	17.0%	29.8%	
Employment						
yes, full–time (*n* = 2,863)	10.1%	16.6%	21.0%	17.2%	35.1%	< 0.001
yes, part–time (*n* = 902)	11.0%	20.7%	27.5%	16.6%	24.2%	
no (*n* = 1,242)	17.3%	17.2%	21.0%	11.8%	32.6%	
Self–assessment of financial situation						
we have enough for everything and we're saving for the future (*n* = 999)	9.6%	14.0%	21.0%	17.2%	38.1%	< 0.001
we have enough for everything without any special sacrifices, but we're not saving for the future (*n* = 977)	9.7%	17.6%	21.3%	17.6%	33.8%	
we live frugally and therefore have enough for everything (*n* = 1,804)	10.3%	17.2%	24.2%	16.1%	32.2%	
we live very frugally to save for major purchases (*n* = 678)	14.4%	20.6%	23.7%	14.3%	27.0%	
we only have enough money for basic needs or we don't have enough money for even the cheapest food (*n* = 547)	23.6%	20.5%	17.0%	10.4%	28.4%	
Respondent's health compared to peers						
definitely better or slightly better (*n* = 1,342)	9.9%	16.7%	18.2%	17.4%	37.8%	< 0.001
the same or hard to say (*n* = 2,323)	8.0%	16.0%	22.5%	16.6%	36.9%	
slightly worse (*n* = 1,003)	19.5%	20.2%	25.9%	14.0%	20.3%	
definitely worse (*n* = 338)	26.4%	23.1%	24.0%	8.6%	17.8%	

A multivariable logistic regression model predicting whether a respondent experienced sleep disturbances daily or almost daily in the 3 months preceding the study yielded a Cox&Snell R2 of 0.050 and a Nagelkerke R2 of 0.096 (Table 3). The Hosmer–Lemeshow goodness-of-fit test yielded a chi-square value of 9.212 with 8 degrees of freedom (*p* = 0.325). Variance inflation factor (VIF) values ranged from 1.07 to 1.90. Tolerance values ranged from 0.53 to 0.93. The receiver operating characteristic (ROC) analysis yielded an area under the curve (AUC) of 0.695 (SE = 0.012, 95% CI: 0.672–0.718, *p* < 0.001). Females had higher odds of reporting sleep disturbances compared to males (aOR: 1.60; 95%CI: 1.33–1.92; *p* < 0.001). Those aged 35–44 years had lower odds of reporting sleep disturbances compared to those aged 45–64 years (aOR: 0.77; 95%CI: 0.61–0.97; *p* < 0.05). When compared to respondents with higher education, those with primary or vocational education (aOR: 1.70; 95%CI: 1.31–2.20; *p* < 0.001), as well as those with secondary education (aOR: 1.29; 95%CI: 1.01–1.64; *p* < 0.05), had higher odds of reporting sleep disturbances. Residents of the largest cities (over 500,000 inhabitants) had higher odds of reporting sleep disturbances daily compared to rural residents (aOR: 1.56; 95%CI: 1.18–2.06; *p* < 0.01). Respondents who worked part-time had a lower odds of reporting sleep disturbances compared to those who did not work (aOR: 0.75; 95%CI: 0.58–0.98; *p* < 0.05). Those with the poorest financial situation had higher odds of reporting sleep disturbances daily compared to those with the best financial situation (aOR: 1.96; 95%CI: 1.43–2.69; *p* < 0.001). Those who rated their health as significantly worse or slightly worse than their peers had higher odds of reporting sleep disturbances daily compared to those in better health than their peers (*p* < 0.001) (Table 3).

**Table 3:** Multivariable logistic regression model predicting whether a respondent experienced sleep disturbances every day or almost every day in the last 3 months preceding the survey (*n* = 5006).

	aOR (95% CI)	*p*
Gender		
male	Reference	Reference
female	1.60 (1.33–1.92)	< 0.001
Age group [years]		
18–24	1.08 (0.80–1.46)	0.598
25–34	0.98 (0.77–1.26)	0.874
35–44	0.77 (0.61–0.97)	< 0.05
45–64	Reference	Reference
Educational level		
primary or vocational	1.70 (1.31–2.20)	< 0.001
secondary	1.29 (1.01–1.64)	< 0.05
higher	Reference	Reference
Place of residence		
rural area	Reference	Reference
city < 100,000	1.08 (0.88–1.33)	0.475
city 100,000–499,000	0.97 (0.74–1.27)	0.825
city >= 500,000	1.56 (1.18–2.06)	< 0.01
Employment		
yes, full-time	0.88 (0.71–1.10)	0.263
yes, part-time	0.75 (0.58–0.98)	< 0.05
no	Reference	Reference
Self-assessment of financial situation		
we have enough for everything and we're saving for the future	Reference	Reference
we have enough for everything without any special sacrifices, but we're not saving for the future	0.92 (0.68–1.25)	0.609
we live frugally and therefore have enough for everything	0.97 (0.74–1.27)	0.805
we live very frugally to save for major purchases	1.20 (0.87–1.64)	0.267
we only have enough money for basic needs or we don't have enough money for even the cheapest food	1.96 (1.43–2.69)	< 0.001
Respondent's health compared to peers		
definitely better or slightly better	Reference	Reference
the same or hard to say	0.81 (0.64–1.02)	0.075
slightly worse	2.10 (1.64–2.68)	< 0.001
definitely worse	2.72 (1.98–3.74)	< 0.001

## 4 Discussion

This nationwide study provides evidence that short sleep duration and sleep disturbances are common among working-age adults in Poland, highlighting sleep health as an underrecognized public health issue. Findings from this study revealed that a significant proportion of working-age adults in Poland (17.9%) reported sleeping less than 7h per night on weekdays. Among working-age adults, almost three times fewer respondents reported sleeping less than 7 hours on weekends (6.5%) compared with weekdays, which may indicate a pattern of compensatory weekend sleep following work-related sleep restriction. Over 70% of respondents reported sleep disturbances in the last 3 months, wherein daily or almost daily sleep disturbances were reported by 12.0% of respondents. There were significant differences in the prevalence of short sleep duration on weekdays by gender, age, educational level, and occupational status. Additionally, health status differentiated the prevalence of short sleep on weekends. All 7 sociodemographic variables included in this study were significantly (*p* < 0.05) associated with reporting sleep disturbances on a daily or almost daily basis. It should be noted that the study population includes both employed and non-employed individuals, which should be considered when interpreting findings in the context of occupational health.

Short sleep duration and sleep disturbances are linked with adverse health events (4, 5, 7, 9). Sleeping at least 7 h per day is necessary for physical and mental functioning. Working-age adults are at higher risk of short sleep on weekdays due to their occupational responsibilities as well as caregiver responsibilities (5, 6, 26). The observed difference between weekday and weekend sleep duration likely reflects a pattern of “social jetlag,” where individuals accumulate sleep debt during working days and compensate during weekends. This phenomenon is commonly linked to work schedules, social obligations, and circadian misalignment, and has been associated with adverse metabolic and mental health outcomes in previous studies (26). Scott et al. in an analysis of 73 million nights reported 20–35 min longer sleep duration on weekends vs. weekdays (26). Between the 1970s and 2000s, the prevalence of short sleep duration increased in Italy and Norway, wherein a decrease was observed in Sweden, the United Kingdom, and the United States (27). In the United States, 33.2% of adults reported short sleep duration in 2020 (28). Benjafield et al. (29), in a group of 852,325,091 adults, reported that the global prevalence of insomnia was estimated at 16.2%. There is limited data on sleep health from Poland (18–20). Zatońska et al. (18) in the PURE Poland cohort study reported that the median sleep duration of women was 30 min longer than that of men. A sleep duration of >8 h was more common in rural than in urban participants (18). Nowicki et al. (19) in the NATPOL study (conducted in 2011) showed that 50.5% of adults in Poland reported sleep complaints (58.9% in women and 41.4% in men) and 60.2% reported difficulties in falling asleep. In this study, 17.9% of working-age adults reported sleeping less than 7 h on weekdays, and 6.5% in weekends. This observation is in line with global data on longer sleep duration on weekends compared to weekdays (26). There is a lack of previously published nationwide data on short sleep duration in Poland, so direct comparisons with other studies are not possible.

In this study, sleep disturbances were defined as experiencing insomnia, trouble falling asleep, or waking up in the middle of the night and having trouble falling back asleep in the last 3 months. Different definitions and measures are used in questionnaire-based studies with self-reported assessments of sleep health (3, 4, 8, 12). We decided to focus on the last 3 months to reduce the risk of recall bias. Sleep disturbances at least once a month were reported by 52% of respondents. This finding is comparable to those reported in the NATPOL study (50.5%) by Nowicki et al. (19). Daily or almost daily sleep disturbances were reported by 12% of respondents. This observation suggests that a significant proportion of working-age adults had sleep disturbances and may require further diagnostics related to sleep health. Insufficient duration and quality of sleep have an impact on personal, social, and economic performance. Sleep health should be prioritized in national health policies.

There were significant differences in sleep duration and sleep disturbances by sociodemographic factors. Males more often reported short sleep duration, which is in line with gender differences in sleep duration reported by Zatońska et al. (18) in PURE Poland. Older respondents (45–64 years) also more frequently reported sleeping less than 7 h per night. This may result from the fact that older people often need less sleep compared to younger people (30). Those with primary or secondary education also more often reported short sleep duration. This may result from the job type related to educational level – e.g., manual workers who start their job earlier (e.g., in a factory or a retail store). Low economic status was also linked to shorter sleep duration. This may indicate the social determinants of sleep health and requires further investigation (31). Full-time employers more often reported short sleep duration on weekdays, whereas unemployed individuals reported short sleep duration on weekends. Occupational duties may affect the sleeping duration on weekdays in full-time employees. Unemployed individuals may not need compensatory sleep on weekends, so shorter sleep duration may be observed in this group. In this study, there was no impact of place of residence on sleep duration. This observation is in contrast with the PURE Poland cohort, where rural inhabitants reported longer sleep duration (18).

In this study, factors associated with daily or almost daily sleep disturbances were also analyzed. Females were more likely to report sleep disturbances. Hormones, biological life cycles of menstruation, and menopause have an impact on the sleep in women and may explain the observed phenomenon (32). Younger adults were less likely to report sleep disturbances. The risk of sleeping problems may increase with age, e.g., due to the physiological changes in the body as well as the risk of chronic conditions that may affect sleep quality (32). Those with primary or vocational education were more likely to report sleep disturbances. We can hypothesize that those with higher education are more likely to adopt sleep hygiene rules and adjust their bedrooms to provide a comfortable environment for sleep (33). Residents of larger cities were more likely to report sleep disturbances that may be linked to environmental factors like exposure to light pollution and noise (34). Respondents who worked part-time had a lower odds of reporting sleep disturbances, which may result from their habits and organization of the day and duties during the day. A poor financial situation was associated with higher odds of reporting sleep disturbances. This observation points out socioeconomic differences in sleep health. We can hypothesize that low financial status is associated with a smaller apartment area, a higher risk of locating in an area exposed to environmental hazards, and a lower possibility of ensuring optimal sleeping conditions. Chronic conditions are common causes of sleeping problems (35). In this study, a worse health status was associated with a higher risk of sleeping problems, which is in line with the previously published data.

The observed associations with education, financial situation, and place of residence suggest that sleep health is shaped by broader social determinants. Individuals with lower socioeconomic status may face environmental and occupational constraints that limit sleep duration and quality, such as shift work, housing conditions, and stress exposure. These findings support the need to consider sleep health within the framework of health inequalities.

The non-significant result of the Hosmer–Lemeshow test suggests that the model predictions are consistent with the observed data, indicating an adequate model fit. The low variance inflation factor (VIF) values indicate that multicollinearity among the predictors was not a concern and did not materially affect the stability of the estimated coefficients. The observed AUC indicates a moderate level of discriminative ability, suggesting that the model was able to distinguish between individuals with and without the outcome better than expected by chance. Although ties in predicted probabilities were observed, they are unlikely to have materially influenced the overall assessment of model performance.

### 4.1 Practical implications

Findings from this study showed that public health strategies in Poland should address sleep health, especially short sleep duration on weekdays among working-age adults. Socio-economic costs of sleep disturbances should be monitored. There is a need to initiate a nationwide debate on sleep health, and the health system should offer targeted actions focused on the diagnosis of sleep disorders. Moreover, educational campaigns on sleep health are needed. This study also provides data on target populations that are highly affected by short sleep duration or sleep disturbances: males, adults aged 45–64 years, those with primary or secondary education, and individuals with poor economic status. Workplace health promotion, occupational safety, and lifestyle medicine interventions are needed to provide sufficient sleep health education.

### 4.2 Limitations

This is a cross-sectional study so data on sleep health were self-declared. Cross-sectional design precludes any inference of causal relationships between variables. Sleep duration was based on questions on average time on going sleep and waking up, without direct monitoring of respondents. Sleep disturbances were assessed using a single self-reported question combining multiple constructs, which may have resulted in misclassification and limited construct validity. The quality of sleep, which is as important as its duration, was also not analyzed. A group of working-age adults was included into this study, but almost one-quarter of them were unemployed, so these results do not reflect the population of employees but working-age adults. An analysis of medical conditions that may affect sleep disturbances was not conducted. Findings from this study can to be generalized to the general population. The relationship between sleep duration and sleep disturbances was not explored and should be addressed in future analyses. Moreover, respondents where not asked about health-related, lifestyle-related and environmental factors that may affect sleep health. Inclusion of both employed and non-employed individuals may have influenced the observed associations, especially those related to work-related factors and sleep patterns.

## 5 Conclusions

This study revealed a relatively high prevalence of short sleep duration on weekdays among working-age adults. Over one-tenth of working-age adults experienced sleep disturbances daily or almost daily that may affect their social and professional roles. Findings from this study underline the need to strengthen public health interventions focused on sleep health. Interventions should be particularly targeted to males, older adults, those with primary or vocational education, and those of poor economic status.

## Data Availability

The data analyzed in this study is subject to the following licenses/restrictions: Dataset used in this study are available from the correspoding author upon reasonable request. Requests to access these datasets should be directed to r.sierpinski@uksw.edu.pl.
